# Artificial intelligence in optimizing antimicrobial therapy for gastro-renal disorders

**DOI:** 10.3389/fcimb.2026.1792361

**Published:** 2026-05-25

**Authors:** Oana Stoia, Paula Anderco, Teona Badiu, Samuel Bogdan Todor, Cristian Ichim

**Affiliations:** Faculty of Medicine, Lucian Blaga University of Sibiu, Sibiu, Romania

**Keywords:** antibiotic treatment, antimicrobial resistance, artificial intelligence, gastrointestinal diseases, renal diseases

## Abstract

Antimicrobial therapy remains central to the management of gastrointestinal and urinary tract infections, yet its effectiveness is increasingly compromised by antimicrobial resistance and antibiotic-induced microbiome disruption. These challenges are particularly pronounced in gastro–renal settings, where recurrent infections, altered drug absorption and impaired renal clearance generate substantial pharmacokinetic variability and narrow therapeutic margins. Empiric, guideline-based regimens may therefore contribute to treatment failure, resistance selection, and disease recurrence. Artificial intelligence (AI) and machine learning offer novel opportunities to optimize antimicrobial therapy by integrating clinical, microbiological and multi-omics data to predict resistance, guide antibiotic selection and dosing, and support antimicrobial stewardship. However, clinical translation remains limited by data heterogeneity, insufficient prospective validation, regulatory constraints, and the need for continued human oversight. This review synthesizes current AI-driven strategies relevant to gastro–renal infections, highlighting shared pathophysiological challenges, practical clinical applications and key limitations. An integrated framework for AI-assisted antimicrobial optimization is proposed to enhance therapeutic efficacy while mitigating antimicrobial resistance and preserving microbiome integrity.

## 1 Introduction

Antimicrobial agents are essential in the treatment of infectious diseases and in enhancing clinical outcomes (Roger, 2024). Their clinical effectiveness, however, is increasingly threatened by the accelerated emergence of antimicrobial resistance (AR), which has become one of the most pressing global public health challenges (Ferrara et al., 2024). Achieving maximum bacterial eradication while limiting the development of resistance depends on a comprehensive understanding of how these drugs interact with pathogenic microorganisms (Veiga and Paiva, 2018; Roger, 2024).

Although pathogenic bacteria are traditionally classified based on phenotypic characteristics such as Gram staining, substantial intra-group heterogeneity exists at both the genetic and functional levels, resulting in marked variability in antimicrobial susceptibility profiles (Rees et al., 2023). This diversity complicates the identification of common molecular targets and the development of antibiotics with universal efficacy (Serral et al., 2021). Coupled with the rapid evolutionary capacity of microorganisms and selective pressure exerted by antimicrobial exposure, these factors have contributed to the widespread emergence of resistance mechanisms affecting nearly all major antimicrobial classes (De Oliveira et al., 2020; Peraman et al., 2021).

The microbiome comprises a complex network of symbiotic, commensal and pathogenic microorganisms occupying diverse niches throughout the human body (Baron et al., 2018). This ecosystem includes bacteria, viruses, archaea and eukaryotic organisms that co-evolve under diverse selective pressures, including pharmacological interventions, dietary patterns, metabolic status, and behavioral factors (Weersma et al., 2020; Wang et al., 2024). Within this shared microbial environment, competitive pressures drive the synthesis of antibiotic molecules and the propagation of AR genes as survival mechanisms (Liu et al., 2025). Consequently, the microbiome functions as a major reservoir of resistance determinants, facilitating horizontal gene transfer to pathogenic bacteria and contributing to the rising incidence of multidrug-resistant infections (Lathakumari et al., 2024; Lertcanawanichakul et al., 2025).

The use of antibiotics has become increasingly problematic as it disrupts the delicate balance of the human gut microbiota (Ramirez et al., 2020). Broad-spectrum antibiotics, while effective against infections, also destroy beneficial bacteria that support gut health (Fan and Pedersen, 2021). Antibiotic-induced dysbiosis initiates a cascade of microbial and immunological disturbances, leading to reduced microbial diversity and the creation of selective niches that favor colonization by resistant organisms (Muteeb et al., 2023; Lathakumari et al., 2024). These effects are particularly relevant in gastrointestinal and renal disorders, where microbiome integrity plays a central role in disease progression, treatment response, and recurrence risk.

Patient-specific pathophysiological states can influence the pharmacodynamic behavior of antibiotics (Phe et al., 2020; Ruiz-Ramos et al., 2023). Accurate recognition of these changes is essential for antibiotic dose optimization, particularly in patients with gastrointestinal dysfunction, renal impairment or critical illness (Koulenti et al., 2025). Variations in fluid balance, tissue perfusion and organ function can substantially alter pharmacokinetic processes, rendering conventional dosing strategies inadequate for achieving therapeutic concentrations in severely ill patients (Shah et al., 2015; Roger, 2024). In this context, inappropriate dosing may not only reduce therapeutic efficacy but also accelerate resistance development and increase toxicity risk.

The remarkable adaptability of microorganisms is mediated by a range of mechanisms, including limitations in drug uptake, target modification or inactivation, biofilm formation, efflux pump activation, enzymatic alterations, metabolic pathway remodeling and genetic mutations (Belay et al., 2024; Elshobary et al., 2025). This multifactorial resistance landscape underscores the limitations of conventional, guideline-based antimicrobial strategies, particularly in complex clinical settings such as gastro-renal disease. As a result, integrated approaches combining antimicrobial stewardship, infection control and individualized treatment strategies have become increasingly necessary (Suruchi et al., 2026). Given the escalating burden of drug-resistant infections and the slow pace of novel antibiotic development, there is an urgent need for innovative, data-driven solutions capable of optimizing antimicrobial therapy while preserving microbiome integrity (Zhao et al., 2025).

Artificial intelligence (AI) and machine learning (ML) have emerged as promising tools capable of addressing these challenges by enabling predictive, personalized and adaptive antimicrobial strategies. While AI-driven approaches have been increasingly explored in the context of antimicrobial resistance, their application within integrated gastro–renal frameworks remain insufficiently characterized, despite the shared microbiome–immune–metabolic axis and the profound pharmacokinetic implications inherent to these conditions. This review aims to address this critical gap by synthesizing current evidence on AI-based predictive models, resistance management strategies and precision treatment approaches tailored to gastrointestinal and renal disorders, highlighting both their clinical potential and the challenges that must be overcome for successful implementation.

## 2 Search strategy

This narrative review was conducted to provide a structured overview of studies exploring the application of AI and ML techniques in the optimization of antimicrobial therapy for gastrointestinal and renal diseases. The objective was to capture experimental, translational and clinical evidence related to resistance prediction, individualized treatment selection and AI-supported antimicrobial stewardship in gastro-renal infections.

Two electronic databases, PubMed and ScienceDirect, were systematically searched to ensure coverage of biomedical, clinical and computational research. The search encompassed publications available up to December 2025, with earlier foundational studies included when they contributed essential mechanistic insights or contextual understanding of antimicrobial resistance or AI-driven methodologies. The search strategy employed a combination of controlled vocabulary terms, where applicable and free-text keywords. These terms were organized into four major thematic groups:

- Antimicrobial treatment and resistance mechanisms (“antimicrobial therapy”, “antibiotic use”, “antibiotic resistance”, “multidrug resistance”, “antimicrobial stewardship”, “therapy optimization”);
- Gastrointestinal diseases and microbiota-related infections (“gastrointestinal disorders”, “Helicobacter pylori”, “Clostridioides difficile”, “gastric infection”, “colitis”, “inflammatory bowel disease”, “gut microbiome”, “intestinal dysbiosis”);
- Renal and urinary tract infections (“renal disorders”, “urinary tract infection”, “UTI”, “pyelonephritis”, “cystitis”, “uropathogenic bacteria”);
- Artificial intelligence and computational methods (“artificial intelligence”, “machine learning”, “deep learning”, “predictive analytics”, “clinical decision support”, “computational intelligence”).

Only English-language, peer-reviewed articles were considered eligible. Included studies comprised *in vitro* and *in vivo* investigations, observational and interventional clinical studies, translational research, and high-quality systematic reviews or meta-analyses, provided they reported outcomes relevant to antimicrobial efficacy, resistance prediction, treatment personalization or AI-assisted clinical decision-making.

Publications were excluded if they consisted solely of conference abstracts without full text, opinion pieces or narrative commentaries lacking analytical depth, case reports without broader translational relevance or studies that did not address antimicrobial therapy, resistance or AI-based analytical frameworks. Studies conducted exclusively in non-human models without clear applicability to human gastrointestinal or renal disease were also excluded.

## 3 Antibiotic resistance

AR has emerged as a critical global health concern, significantly compromising our ability to treat bacterial infections effectively (Ahmed et al., 2024). Although the phenomenon of resistance has been recognized for decades by experts in microbiology and infectious diseases, dating back to Sir Alexander Fleming’s early warnings regarding penicillin underdosing, its magnitude and clinical impact have only recently gained broader recognition at the public health level (Seaton, 2025). As a result, numerous infectious agents that were once easily controlled using multiple antibiotic classes now exhibit resistance to most, and occasionally all, available drugs (Kahn, 2017; Hutchings et al., 2019).

AR arises through several well-characterized biological strategies, four of which predominate in clinical settings:

- enzymatic inactivation of antibiotics, such as β-lactamase production;
- impaired intracellular drug accumulation resulting from reduced membrane permeability or enhanced efflux pump activity;
- biofilm-associated protection mechanisms;
- structural or functional modifications of antibiotic target sites, including alterations in essential proteins (Blair et al., 2015; Munita and Arias, 2016; De Oliveira et al., 2020).

These mechanisms often coexist within the same organism, contributing to multidrug resistance and further complicating therapeutic decision-making.

**Figure 1:**
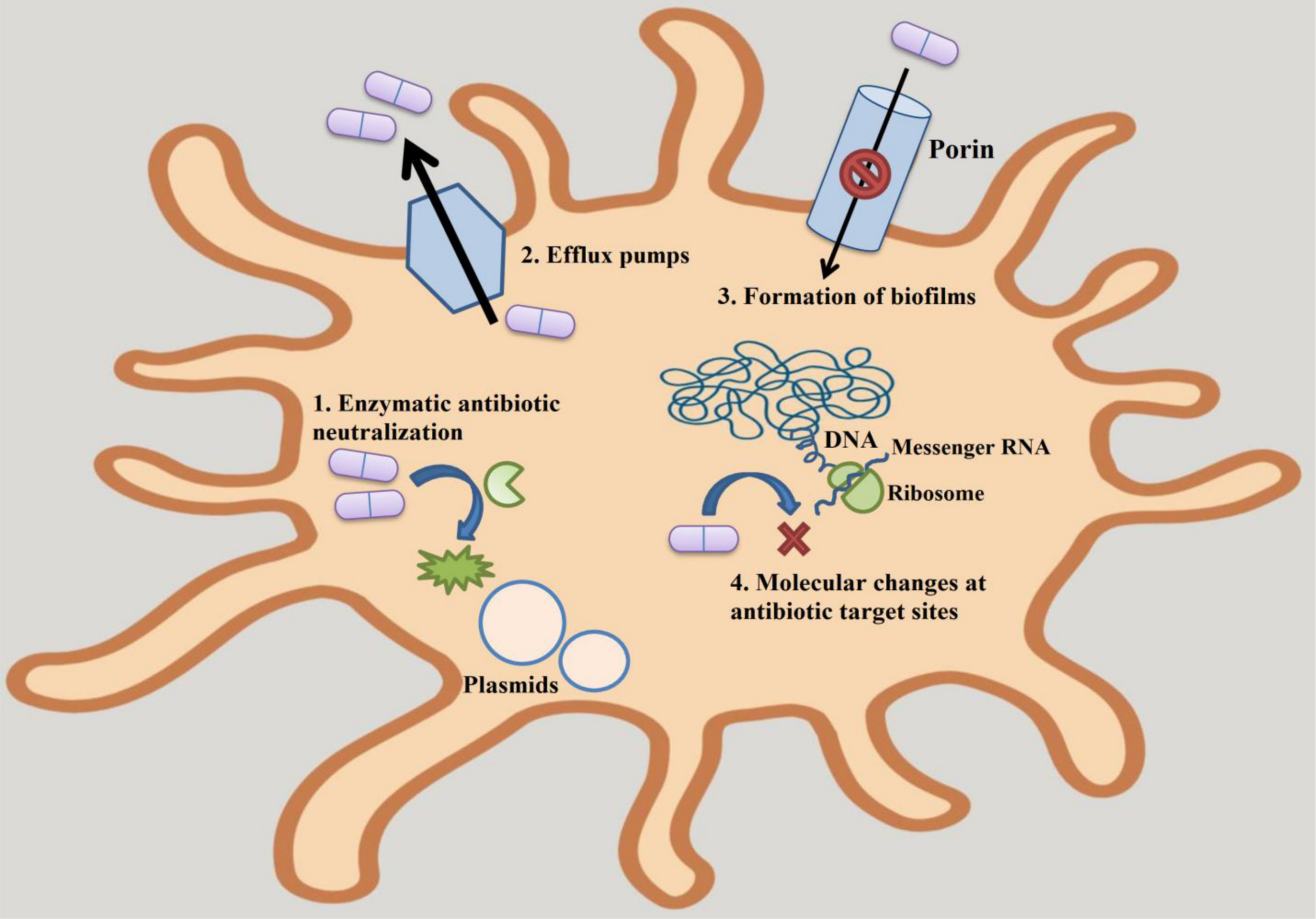
Primary biological mechanisms leading to AR.

Plasmids and other mobile genetic elements play a central role in the dissemination of resistance traits and the evolutionary adaptation of bacterial populations (Domingues et al., 2025). Plasmids are extrachromosomal DNA elements that frequently harbor antibiotic resistance genes and can be transferred between bacteria through horizontal gene transfer mechanisms, including transformation, transduction and conjugation (Darby et al., 2023; Branda and Scarpa, 2024). This genetic mobility facilitates the rapid spread of resistance across bacterial species and ecological niches, amplifying the clinical and epidemiological impact of AR. An overview of the principal biological mechanisms involved in antibiotic resistance is illustrated in **Figure 1**.

Bacterial infections caused by multidrug-resistant organisms represent a major and growing challenge for healthcare systems worldwide. The increasing prevalence of AR has reduced the efficacy of existing antibiotics, highlighting the need for alternative therapeutic approaches (Chiş et al., 2022; Sahel et al., 2024; Kashyap et al., 2025). Furthermore, sepsis represents a major cause of hospitalization in intensive care settings and studies have shown that the prompt initiation of appropriate antibiotic therapy may enhance clinical outcomes (Seymour et al., 2017; Rhee et al., 2020). The failure of antimicrobial therapy is influenced by bacterial resistance, most notably as a consequence of antibiotic misuse, which acts as a key driver in the development of resistance (Roger et al., 2025).

The development of novel antibiotics capable of circumventing established resistance mechanisms is essential for sustaining effective antimicrobial therapy. Nevertheless, broad-spectrum strategies that indiscriminately eradicate bacterial populations pose significant risks, particularly given the critical role of the human microbiome in maintaining metabolic, immunological and barrier functions (Maier et al., 2021; Peraman et al., 2021; Rees et al., 2023). Disruption of commensal microbial communities not only predisposes patients to secondary infections but may also facilitate the emergence and persistence of resistant organisms (Khosravi and Mazmanian, 2013).

Continued progress in combating AR therefore depends on sustained investment in antimicrobial research and development, alongside the implementation of innovative therapeutic and preventive strategies. These include targeted antimicrobial approaches, microbiome-preserving interventions and optimized stewardship programs designed to balance therapeutic efficacy with ecological considerations (Jacobowski et al., 2025; Lucero-Prisno et al., 2025). As the global burden of antibiotic resistance continues to rise, integrated strategies that combine biological insight with technological innovation will be essential to preserve the effectiveness of existing antibiotics and improve patient outcomes (Peraman et al., 2021; Lathakumari et al., 2024; Kashyap et al., 2025).

## 4 Therapeutic interventions for gastrointestinal diseases

Gastrointestinal (GI) disorders encompass a range of conditions, including inflammatory diseases, potential malignancies, peptic ulcer disease and irritable bowel syndrome (Jabłońska and Mrowiec, 2023). In recent years, the prevalence of GI diseases has increased globally, with many conditions characterized by chronic or recurrent evolution and a substantial dependence on antimicrobial therapy, which complicates long-term disease management (Jiang et al., 2025).

The GI tract is a highly complex system where the host immune defenses engages with a wide range of beneficial and harmful microorganisms (Layunta et al., 2024). To maintain intestinal homeostasis, immune responses must be precisely regulated to control pathogenic microorganisms while preserving commensal flora, as excessive or prolonged antimicrobial exposure may disrupt this balance (Chung and Raffatellu, 2019). GI mucosal integrity is a key determinant of intestinal homeostasis and is maintained through a balance between destructive stimuli that cause mucosal injury and protective factors that promote tissue repair (Vancamelbeke and Vermeire, 2017; Gieryńska et al., 2022). When this balance is disturbed, susceptibility to infection increases and antimicrobial interventions become more frequent, contributing to cumulative antibiotic exposure (Oncel and Basson, 2022; Otte et al., 2023).

Current therapeutic approaches for GI disorders encompass drug-based treatments, endoscopic and interventional procedures, and surgical management (Singh et al., 2022; Suri et al., 2024). Pharmacological therapy remains the primary option for most patients due to its effectiveness (Ohia et al., 2022). GI drug delivery offers several advantages, including non-invasive administration, improved patient comfort, reduced healthcare costs, and ease of use (Chu and Traverso, 2022). Within this context, antibiotics represent a major therapeutic class, widely prescribed for both acute and chronic GI conditions with an infectious component, such as cholangitis, *Helicobacter pylori* (*H. pylori*) infection, infectious colitis, and diverticular disease (Elbehiry et al., 2024).

*H. pylori* infection is a key etiological factor in chronic gastritis, peptic ulcer disease, and gastric carcinoma and has been classified as a Group 1 carcinogen by the International Agency for Research on Cancer (Salvatori et al., 2023; Wu et al., 2025). Eradication therapy is therefore a cornerstone of disease prevention and management, as bacterial elimination reduces ulcer recurrence and lowers gastric cancer risk (Baj et al., 2020; Wang et al., 2026). Conventional eradication regimens typically consist of a proton pump inhibitor combined with two antibiotics, administered either sequentially or concomitantly for durations ranging from five days to two weeks, depending on clinical context (Hussein et al., 2022; Anand and Tharmaraj, 2025). However, increasing resistance to commonly used antibiotics and high rates of treatment failure have progressively reduced eradication success, leading to repeated treatment courses and increased antimicrobial exposure (Jing et al., 2025; Wang et al., 2026).

Infectious and inflammatory colitis represent another major indication for antimicrobial use in GI practice. Colitis is suspected in patients presenting with diarrhea associated with abdominal pain, fecal urgency, tenesmus or dysentery, with diagnosis confirmed by endoscopic evidence of colonic inflammation (Adams and Close, ). Supportive findings include elevated fecal inflammatory markers, such as calprotectin or lactoferrin (Johnson et al., 2020; Langhorst et al., 2020). Acute colitis is most commonly of infectious origin, whereas chronic forms are more frequently associated with inflammatory bowel disease, although recurrent *Clostridioides difficile* infection must also be considered (Iqbal and DuPont, 2021).

*Clostridioides difficile* infection is a leading cause of antibiotic-associated colitis and represents a paradigmatic example of the relationship between antimicrobial exposure and dysbiosis (Guh et al., 2020; Wang et al., 2024). The disease spectrum ranges from mild diarrhea to severe, life-threatening complications, with pseudomembranous colitis representing the most severe form (Leffler and Lamont, 2015; Wang et al., 2024; Stămăteanu et al., 2025). Antibiotic-induced disruption of the gut microbiome plays a central role in both disease onset and recurrence, making management particularly challenging in patients requiring repeated or prolonged antimicrobial therapy (Ramakrishna and Patankar, 2023; Cusumano et al., 2025).

Other GI conditions, including Crohn’s disease and diverticular disease, frequently require antimicrobial treatment for disease complications rather than primary infection. Crohn’s disease is a chronic inflammatory disorder driven by dysregulated immune responses in genetically susceptible individuals (Cockburn et al., 2023; Dotlacil et al., 2023; Elsayed et al., 2026). Although not primarily infectious, antibiotics are commonly used to treat secondary complications, contributing to cumulative antibiotic exposure. Similarly, diverticulitis ranges from uncomplicated inflammation to complicated disease forms requiring antimicrobial therapy, with a subset of patients developing smoldering disease characterized by persistent symptoms and inflammatory changes (Munie and Nalamati, 2018; Peery et al., 2019; Kaise et al., 2020; Peery et al., 2021). Management strategies often combine dietary measures, antibiotics, and anti-inflammatory agents, with surgery reserved for refractory cases (Crowe et al., 2011; Schembri, 2017; Piscopo and Ellul, 2020; Kishnani et al., 2022; Urquhart et al., 2024).

Antibiotic therapy in GI diseases is frequently limited by adverse drug reactions. Beta-lactams are most commonly implicated, followed by fluoroquinolones and macrolides (Kokado et al., 2019). Cutaneous reactions occur in approximately 1–5% of patients and are particularly associated with aminopenicillins, vancomycin, and trimethoprim/sulfamethoxazole (Urakami and Matono, 2025). Certain antibiotics, including macrolides, quinolones and trimethoprim/sulfamethoxazole, also increase the risk of rhabdomyolysis, especially when co-administered with statins, necessitating temporary treatment modification (Chuma et al., 2022; Samura et al., 2022).

In routine clinical practice, antimicrobial therapy for GI infections is frequently initiated empirically, based on the most likely causative organisms associated with specific anatomical sites (Eyler and Shvets, 2019; Edwards et al., 2021). **Table 1** summarizes common GI infections, their predominant pathogens, and recommended empirical treatment regimens (Bardsley et al., 2020; Glassner et al., 2020; Iqbal and DuPont, 2021; Johnson et al., 2021; Peery et al., 2021; Sun et al., 2023; Chey et al., 2024; Cymbal et al., 2024; Triantafillidis et al., 2024). While empiric strategies are often necessary, they may contribute to inappropriate antibiotic selection and further resistance development, particularly in recurrent or chronic disease.

**Table 1:** Prevalent bacterial pathogens and initial antimicrobial management.

Clinical condition	Predominant causative organisms	Recommended initial therapy
Antibiotic-associated colitis	*Clostridioides difficile*	Fidaxomicin or oral Vancomycin
*H. pylori* infection	*H. pylori*	Clarithromycin plus Amoxicillin with a PPI or Metronidazole-based regimen
Infectious gastroenteritis	*Campylobacter, Salmonella, Shigella*	Azithromycin or Ciprofloxacin
CD–related complications	Secondary bacterial infection	Ciprofloxacin and/or Metronidazole
Acute diverticular disease	Polymicrobial intestinal flora	Metronidazole combined with Ciprofloxacin

Adjunctive approaches, including probiotic supplementation, have been shown to reduce *H. pylori* colonization and improve eradication rates when used alongside antibiotic therapy. Probiotics may also attenuate antibiotic-induced dysbiosis and gastrointestinal adverse effects, supporting their role as complementary interventions in GI antimicrobial management (Koga, 2022).

## 5 Therapeutic interventions for renal diseases

Urinary tract infections (UTIs) caused by bacteria represent a major global health burden and are often managed with empirical antibiotic therapy in the absence of microbiological identification (Farag et al., 2024). This reliance on empiric treatment is particularly relevant in renal disease, where delayed therapy may worsen outcomes, but inappropriate antibiotic selection may increase resistance and toxicity risks (Bassetti et al., 2020; Zasowski et al., 2020). The development and progression of UTIs is a multifactorial process involving compromised host immune defenses, enhanced microbial pathogenicity, and underlying anatomical abnormalities of the urogenital tract (Teh, 2022). When immune defenses are compromised, pathogenic or opportunistic bacteria are able to attach to the urinary epithelium, multiply, and persist, leading to infection (Farag et al., 2024). The etiology of UTIs includes both exogenous and endogenous origins, with the majority of infections linked to exogenous factors (Yu et al., 2020).

Moreover, pathogenic microorganisms may be introduced into the urinary tract via the urethra during catheterization procedures, potentially resulting in iatrogenic urinary tract infections (Farag et al., 2024). UTIs are classified based on the anatomical site involved into upper tract conditions, such as pyelonephritis and ureteritis, and lower tract conditions, including cystitis and urethritis (Teh, 2022). From a therapeutic perspective, this anatomical distinction primarily guides antibiotic selection, route of administration, and treatment duration rather than diagnostic classification alone.

Lower UTIs typically manifest with symptoms including dysuria, hematuria, inappropriate urination, urinary incontinence, and urine dribbling (Lajiness and Hintz, 2020). Diagnostic evaluation typically integrates clinical assessment with hematobiochemical analyses, urinalysis and sediment examination, as well as imaging techniques, including radiography and ultrasonography (Scarborough et al., 2020; Barot et al., 2022). However, in routine clinical practice, antimicrobial therapy is often initiated before complete diagnostic clarification, reinforcing the importance of selecting agents with an appropriate balance between efficacy and safety.

The efficacy of standard antibiotic treatments for UTIs is increasingly compromised by antimicrobial resistance and poor patient adherence. While UTIs are primarily attributable to Enterobacteriaceae and other Gram-negative uropathogens, resistance to guideline-endorsed first-line antimicrobial agents has risen substantially, with statistically significant increases in *Escherichia coli* (*E. coli*) resistance to nitrofurantoin and trimethoprim–sulfamethoxazole documented between 2003 and 2012 (Sanchez et al., 2016; Goodlet et al., 2019).

In numerous countries, *E. coli* isolates causing severe human infections frequently exhibit resistance to third-generation cephalosporins, necessitating increased use of the limited remaining antimicrobial options, including carbapenems (Feng et al., 2022; Aljohni et al., 2025). Such resistance poses serious public health concerns; a systematic review by the World Health Organization demonstrated that patients infected with third-generation cephalosporin–resistant *E. coli* experienced a twofold increase in all-cause mortality, bacterium-attributable mortality and 30-day mortality relative to those with infections due to susceptible isolates (Kahn, 2017).

Although fluoroquinolones and β-lactam antibiotics have historically been utilized as alternative therapeutic options for UTIs, declining susceptibility among uropathogens, particularly to fluoroquinolones, has raised concerns regarding their continued effectiveness (Stapleton et al., 2020; García-Meniño et al., 2024). Moreover, antimicrobial stewardship principles restricting the use of broad-spectrum antibiotics that are first-line treatments for prevalent inpatient infections, including pneumonia and intra-abdominal infections, to preserve their antimicrobial utility. As a result, therapeutic options for UTIs are increasingly constrained, emphasizing the need for optimized antibiotic selection and duration (Goodlet et al., 2019).

Renal function frequently necessitates antibiotic dose modification based on creatinine clearance, and some agents are contraindicated in this context. Tetracycline should be avoided due to its potential to aggravate renal dysfunction. Nitrofurantoin is ineffective in this setting because it is not adequately secreted into the urinary collecting system and is associated with an increased risk of peripheral neuropathy in patients with reduced estimated glomerular filtration rate (eGFR). Aminoglycosides may rapidly accumulate, raising the risk of oto- and nephrotoxicity. Additionally, co-trimoxazole may cause an approximate 10% increase in serum creatinine concentrations without a corresponding change in eGFR (Eyler and Shvets, 2019; Edwards et al., 2021). These pharmacokinetic and safety constraints substantially complicate antibiotic selection in patients with renal impairment and increase the risk of adverse outcomes if dosing is not carefully adjusted. **Table 2** outlines common UTIs and their recommended first-line antibiotic treatments (Kahn, 2017; Eyler and Shvets, 2019; Goodlet et al., 2019; Pistolesi et al., 2019; De Oliveira et al., 2020; Rhee et al., 2020; Scarborough et al., 2020; Yu et al., 2020; Edwards et al., 2021; Nel Van Zyl et al., 2022; Teh, 2022; Farag et al., 2024; Lathakumari et al., 2024; Nelson et al., 2024; Roger, 2024; Borgert et al., 2025; Aslan et al., 2026).

**Table 2:** Etiological agents and first-line antibiotic management of UTIs.

Clinical condition	Predominant causative organisms	Recommended initial therapy
Complicated UTIs	*Klebsiella, E. coli*	Cephalosporins or fluoroquinolones
Uncomplicated lower UTIs	*E. coli*	Nitrofurantoin or fosfomycin
Recurrent UTIs	*E. coli*	Nitrofurantoin (prophylaxis)
Asymptomatic bacteriuria during pregnancy	*E. coli*	Nitrofurantoin or amoxicillin
Acute bacterial prostatitis	*E. coli*	Ciprofloxacin or levofloxacin
Chronic Prostatitis	*E. coli*	Fluoroquinolones
Acute Pyelonephritis	*Proteus, E. coli*	Ceftriaxone or ciprofloxacin
Chronic Pyelonephritis	Gram-negative bacteria	Fluoroquinolones
Emphysematous pyelonephritis	*E. coli*	Carbapenems
Post-streptococcal glomerulonephritis	β-hemolytic *Streptococcus*	Penicillin
Renal Abscess	Mixed bacterial flora	Cephalosporins plus metronidazole

The likelihood of infection increases in proportion to the disruption of the patient’s normal GI flora. Antimicrobial agents such as clindamycin, fluoroquinolones, second- and third-generation cephalosporins and co-amoxiclav being associated with a higher risk (Edwards et al., 2021). This highlights the close interdependence between gastrointestinal microbiome disruption and renal infectious risk, particularly in patients exposed to repeated antibiotic courses. Specific probiotic and dietary nutrients interventions exhibit considerable potential in modulating the gut microbiota and decrease AR bacterial colonization. Additionally, therapeutic strategies that involve phages or adjuvants with antibiotics may enhance antimicrobial longevity by attenuating AR mechanisms and interrupting routes of bacterial transmission (Ding et al., 2023).

The development of innovative alternative therapeutic modalities, including nanoparticle-based systems, vaccines, monoclonal antibodies and the identification of novel antimicrobial targets to support antibiotic discovery, is urgently required to overcome certain mechanisms of resistance. Equally important are rational antibiotic prescribing, individualized dose adjustment, and optimized treatment duration to minimize collateral microbiome damage while preserving therapeutic efficacy (Ding et al., 2023).

## 6 Artificial intelligence–driven approaches to treatment optimization

Over the past decade and a half, the pipeline for new antibiotic development has shown significant weaknesses (Theuretzbacher et al., 2023). Addressing this escalating issue requires robust antimicrobial stewardship strategies (Bilal et al., 2025). Historically, laboratory and clinical datasets were frequently incomplete or underused due to their large volumes, complexity, insufficient awareness, and the lack of standardized collection systems. In recent years, however, the increasing availability of electronic health records integrating clinical, microbiological, and laboratory data has created new opportunities for data-driven optimization of antimicrobial therapy (Macesic et al., 2017).

ML applies sophisticated techniques to generate evidence-based predictions and support clinical decision-making through the analysis of laboratory and clinical data (Bilal et al., 2025). AI further improves this process by quickly evaluating large volumes of information and generating logical conclusions. Robust AI algorithms can compensate for human limitations, including procedural non-adherence, fatigue and biases arising from hierarchical cultural influences. Together, AI and ML offer substantial potential to enhance research efficiency in addressing complex health challenges such as AR (Rajpurkar et al., 2022; Bilal et al., 2025).

**Figure 2:**
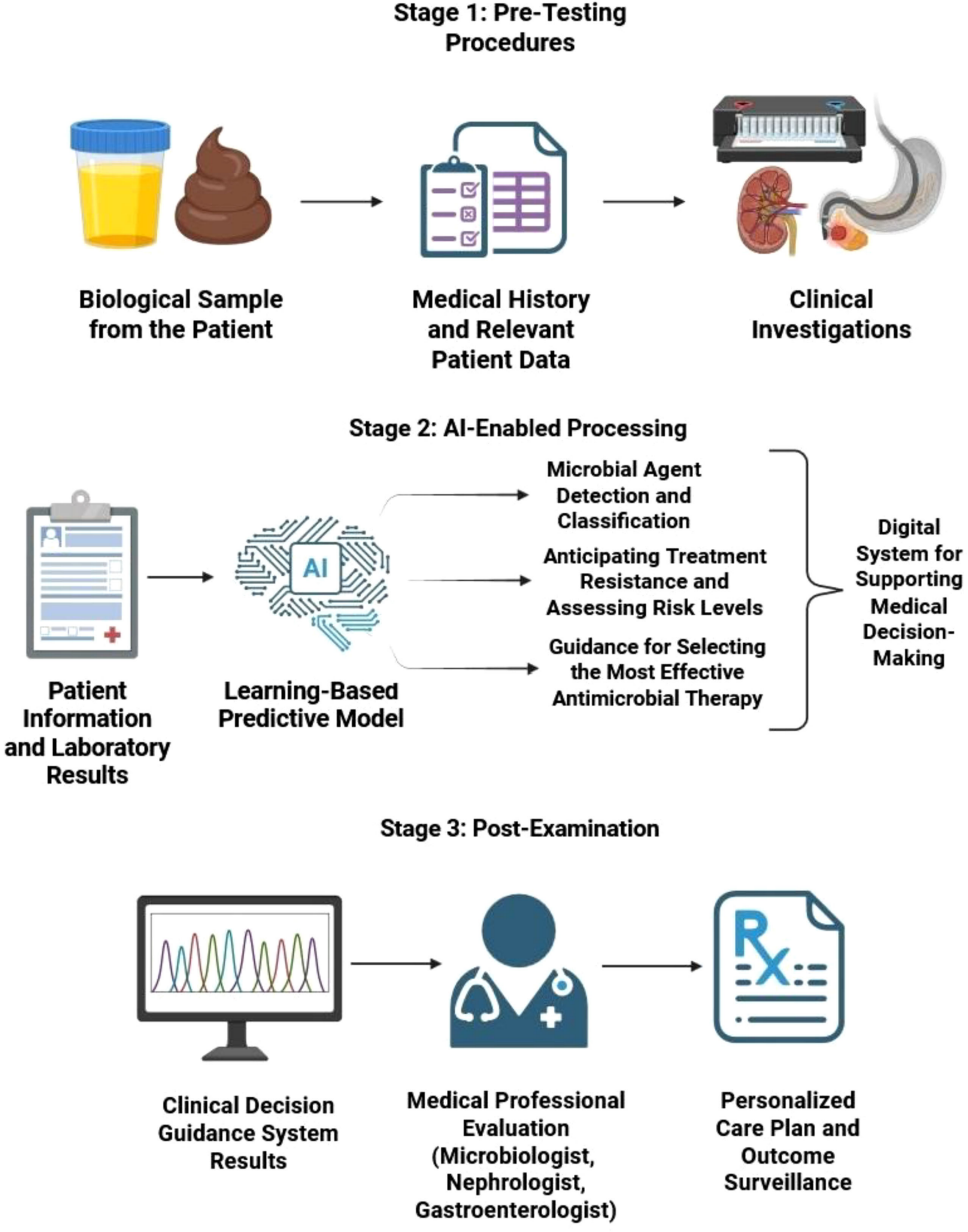
Conceptual framework of artificial intelligence–assisted antimicrobial optimization in gastrointestinal and renal disorders.

AI is a key tool in addressing AR and enhancing treatment effectiveness in patients with gastro-renal conditions. By processing and interpreting data, AI can forecast resistance development, model how microorganisms evolve, track patient responses to therapy, recommend combination treatments, and tailor treatment strategies to individual needs (Johnson et al., 2021). These capabilities are particularly relevant in gastro-renal disease, where pharmacokinetic variability, microbiome disruption, and recurrent antibiotic exposure complicate conventional treatment algorithms. The conceptual workflow of AI-assisted antimicrobial optimization in gastro-renal disorders is illustrated in **Figure 2**.

Initially, AI facilitates large-scale data analysis by examining extensive datasets to detect patterns and identify resistant microorganisms. By handling extensive data efficiently, AI algorithms can detect hidden associations and clarify the causes of resistance development (Pourhajibagher et al., 2024). ML and AI models enable the systematic analysis of complex datasets to predict patients who are likely to develop AR or are at increased risk (De La Lastra et al., 2024). Such predictive capacity supports more rational antibiotic selection and timing, with potential implications for both efficacy and stewardship.

Recent clinical validation studies have shown that ML models trained on electronic medical records can predict antibiotic resistance in urinary tract infections before susceptibility results are available, supporting prototype clinical decision-support outputs for empiric antibiotic selection (Lewin-Epstein et al., 2021; Yang et al., 2023; İlhanlı et al., 2024). In addition, studies using structured susceptibility and patient-variable data from clinical E. coli isolates demonstrated that Random Forest–based models can accurately identify multidrug-resistant patterns, highlighting the feasibility of real-world ML deployment for AMR surveillance and stewardship support (Harris, 2023; Tolan et al., 2025). Representative AI-driven models relevant to gastrointestinal and renal antimicrobial optimization are comparatively summarized in **Table 3**.

**Table 3:** Comparative overview of validated and clinically implemented AI models for antimicrobial optimization in gastrointestinal and renal disorders.

Model and data input	Output	Clinical setting and validation	Development stage	Study
Bayesian model-informed precision dosing using renal function parameters, dosing history and serum vancomycin concentrations	Individualized area-under-the-curve–guided dose adjustment	Hospitalized adults; retrospective comparative cohort	Clinically implemented decision-support system	(Hall et al., 2024)
Population pharmacokinetic model with Bayesian estimation using dialysis parameters, clearance data and measured drug levels	Patient-specific exposure prediction	Hemodialysis population; development cohort with validation dataset	Clinically applicable pharmacokinetic model	(Oda et al., 2023)
Regularized logistic regression applied to electronic health records including laboratory data, vital signs and antimicrobial exposure	Daily predicted probability of Clostridioides difficile infection	Academic hospital; prospective pre–post implementation study	Implemented stewardship intervention	(Tang et al., 2025)
Gradient boosting model trained on clinical, laboratory and microbiological variables	Probability of antibiotic necessity or appropriateness	Internal medicine wards; internal cross-validation	Proof-of-concept predictive model	(Radakovich et al., 2025)
Meta-analysis of machine learning models including random forests, gradient boosting and neural networks applied to heterogeneous clinical datasets	Pooled predictive performance metrics	Predominantly inpatient settings; systematic review and meta-analysis	Evidence synthesis	(Pinto et al., 2025)
Meta-analysis of multiple machine learning approaches applied to electronic health record and microbiological data	Pooled area under the curve, sensitivity and specificity	Mixed inpatient settings; systematic review and meta-analysis	Evidence synthesis	(Pennisi et al., 2025a)
Narrative review of decision trees, random forests, gradient boosting and neural networks	Conceptual applications in resistance prediction and personalized antibiograms	Various healthcare settings; narrative synthesis	Conceptual overview	(Pennisi et al., 2025b)
Review of machine learning–enhanced pharmacokinetic modeling using therapeutic drug monitoring data	Framework for dose optimization strategies	Hospital-based therapeutic drug monitoring; narrative review	Translational framework	(Varela-Rey et al., 2024)

These computational approaches also contribute to antibiotic discovery, drug repurposing, and the identification of synergistic therapies through high-throughput screening of chemical compounds against AR-associated pathogens (Vamathevan et al., 2019). Additionally, ML/AI tools facilitate the surveillance of antimicrobial resistance gene dissemination, risk assessment, public health monitoring, and the identification of novel diagnostic and therapeutic strategies (De La Lastra et al., 2024). Commonly employed ML/AI techniques include supervised and unsupervised learning algorithms, deep learning (DL), reinforcement learning, and related architectures (Shaik et al., 2022; Bilal et al., 2025).

Supervised learning algorithms rely on labeled datasets, such as patient clinical records and microbial genomic data, to generate predictions and support decision-making. For instance, supervised ML approaches have identified genetic traits associated with antibiotic susceptibility in *E. coli* across multiple sequence types, providing insights into the evolutionary dynamics and dissemination of these lineages within susceptible populations (Shaik et al., 2022). Unsupervised learning algorithms function without predefined labels, enabling the autonomous identification of inherent patterns and clusters within complex datasets (Zhang et al., 2020). This capability has facilitated the detection of previously unrecognized associations within microbial communities, including the co-occurrence of antibiotic and metal resistance traits in *Salmonella enterica* (Fenske and Scaria, 2021; Asnicar et al., 2024).

AI also plays an important role in genomic analysis by enabling high-resolution characterization of microbial genomes and the identification of genetic determinants of resistance. Through the analysis of resistant isolate genomes, AI algorithms can detect key mutations or resistance markers, thereby informing targeted therapeutic strategies and preventive interventions (Johnson et al., 2021). The selection of ML and AI methodologies is guided by the nature of the data and the objectives of the analysis, with numerous specialized algorithms offering distinct advantages and limitations beyond these commonly applied techniques (Bilal et al., 2025).

Another critical application of AI involves the development of predictive models that integrate environmental variables, microbial species characteristics, and treatment protocols to anticipate resistance emergence. By combining diverse data streams, ML-based models can estimate resistance risk under different clinical and environmental conditions and assist clinicians in optimizing treatment and prevention strategies (Rajpurkar et al., 2022; Pourhajibagher et al., 2024).

An important feature of this advancement is the use of omics data, including genomics, transcriptomics, proteomics, and metabolomics, providing a broad, multi-layered understanding of bacterial activity (Sanches et al., 2024). This integrative approach has improved the characterization of persister cell formation and survival mechanisms, facilitating the identification of biomarkers associated with antimicrobial tolerance and persistence (Picard et al., 2021).

The identification of such biomarkers is critical for discriminating persistent bacterial strains from non-persistent ones, thereby enabling earlier detection and more targeted treatments. ML algorithms are central to the analysis of these large-scale, complex datasets, uncovering subtle patterns and correlations that are often undetectable by conventional analytical methods (Niu et al., 2024; Yuan et al., 2024).

Training DL algorithms on integrated genomic, proteomic, and metabolic profiles enables the prediction of persister cell formation across varying environmental contexts, such as nutrient deprivation, antibiotic challenge, and host immune modulation (Yakobi and Nwodo, 2025). Integrating predictive models allows researchers to simulate bacterial responses under varying conditions, improving mechanistic understanding and potential mitigation strategies (Grapov et al., 2018).

AI-driven methods have been important in creating tools that allow real-time detection technologies for persister cells. Case studies conducted in both clinical and laboratory settings have demonstrated the utility of these approaches. Specifically, ML models have been employed to imaging data to identify persister cells within biofilms or infection sites (Rashid and Kausik, 2024; Yakobi and Nwodo, 2025).

AR prediction can be conducted at both the phenotypic and molecular levels, each requiring distinct datasets and modeling approaches (Ali et al., 2023). Phenotypic prediction relies primarily on antimicrobial susceptibility testing data, including minimum inhibitory concentration values or zone-of-inhibition measurements, together with patient demographic information such as treatment outcomes, underlying health conditions, age and sex, clinical specimen types, and microbial species identification (Yamin et al., 2023). In contrast, molecular prediction depends on genetic information derived from whole-genome or metagenomic sequencing data (Khaledi et al., 2020).

Treatment efficacy can be predicted based on the presence or absence of resistance determinants. Environmental factors, including temperature and humidity, also affect resistance spread and can be integrated into prediction models (Endale et al., 2023).

Despite the AI-enabled phage therapy systems offer promising opportunities, several challenges must be overcome to ensure their safe and effective clinical application. Regulatory approval represents a major barrier, as automated systems for compounding and delivery must comply with rigorous safety requirements, including ISO 13485 certification, adherence to Good Manufacturing Practices and specific approvals from regulatory authorities such as the FDA or EMA for both technological and therapeutic elements (Mohammadi et al., 2025). These regulatory steps could significantly extend the time required for clinical implementation (Rahimian, 2025).

A further key consideration is achieving an appropriate balance between automation and clinical oversight. Although the system is designed to operate independently, involving clinicians during the early stages of implementation may be necessary to monitor treatment decisions and intervene when required. This approach can help reduce risks until the system proves reliable across different patient populations (Rahimian, 2025).

Variability in human immune responses must be considered by the system. Furthermore, existing phage banks may not include effective options for all infections, especially those involving rare or newly emerging pathogens (Hibstu et al., 2022; Hitchcock et al., 2023). Interindividual variability in phage pharmacokinetics is influenced by factors such as microbiome composition, comorbidities and immune system (Peez et al., 2025). As a result, AI models may need to include personalized adjustment mechanisms to tailor dosing and phage selection, increasing the complexity of system design and validation (Nang et al., 2023).

This transition is highly impactful, as it concurrently enhances clinical outcomes, promotes workforce efficiency and sustainability, and creates opportunities for innovation and cost-effective healthcare delivery (Rahimian, 2025).

## 7 Limitations and ethical considerations of AI in antimicrobial optimization

Despite the substantial potential of AI-driven antimicrobial prediction systems, an overly optimistic or uncritical implementation may introduce significant clinical and methodological risks. The performance of ML models is inherently dependent on the quality, representativeness and completeness of training datasets. Electronic health records and microbiological databases often contain missing values, inconsistent coding practices, sampling biases and local epidemiological patterns that may not generalize across institutions or geographical regions. Consequently, predictive models developed in single-center settings may demonstrate reduced external validity when deployed in broader clinical environments.

Overreliance on algorithmic outputs for resistance prediction or empiric therapy selection may also risk reinforcing existing prescribing biases or perpetuating historical antimicrobial usage patterns embedded within the training data. If stewardship practices were suboptimal during data collection, AI systems may inadvertently replicate and amplify these patterns rather than correct them.

Furthermore, resistance prediction models frequently operate on probabilistic outputs rather than deterministic results. While high predictive accuracy may be achieved statistically, even a small rate of false susceptibility predictions could result in inappropriate antibiotic selection in critically ill patients, potentially worsening outcomes. In high-stakes settings such as sepsis or severe pyelonephritis, the clinical tolerance for algorithmic error remains extremely low.

The use of AI in novel antimicrobial discovery and susceptibility forecasting also raises concerns regarding reproducibility and biological interpretability. Complex deep learning architectures may function as “black-box” systems, limiting mechanistic insight into the biological drivers of resistance. This opacity may reduce clinician trust and complicate regulatory evaluation.

Excessive dependence on AI-driven antimicrobial optimization could inadvertently weaken clinical reasoning skills or diminish microbiological expertise if decision-support tools are adopted without structured oversight and continuous validation. Therefore, AI systems must be positioned as augmentative tools that complement, rather than replace, clinical judgment and microbiological confirmation.

## 8 Conclusions

Gastrointestinal and renal infectious disorders represent a major and increasingly complex clinical challenge, driven by chronicity, recurrent antibiotic exposure, and the accelerating burden of antimicrobial resistance. In these settings, effective antimicrobial therapy requires not only pathogen eradication but also careful consideration of host-specific factors, microbiome integrity, and pharmacokinetic variability.

This review underscores the limitations of conventional, guideline-based antimicrobial strategies when applied to heterogeneous gastro-renal patient populations and highlights the central role of microbiome disruption in resistance development and treatment failure.

Artificial intelligence and machine learning represent a promising analytical framework that may support antimicrobial optimization by enabling resistance prediction, individualized antibiotic selection, and integration of clinical and multi-omics data. When applied as decision-support tools, these approaches have the potential to strengthen antimicrobial stewardship while preserving therapeutic efficacy.

However, the clinical implementation of AI-driven strategies remains constrained by regulatory requirements, limited prospective validation, and the need for ongoing human oversight. As such, AI should complement, rather than replace, clinical expertise and requires continued oversight.
